# Culture‐Negative Infective Endocarditis Presenting With Cardio‐Hepatorenal Syndrome in a 21‐Year‐Old Patient: A Case Report of Diagnostic Workup and Management

**DOI:** 10.1155/cric/7676322

**Published:** 2026-03-09

**Authors:** Jana Kotaich, Safaa Ghanem, Nour Mina, Rayyan El Saleh, Rim Awada, Said Chaaban, Karim Jammal

**Affiliations:** ^1^ Department of Internal Medicine, MEDICA Research Investigation, Hadath, Lebanon; ^2^ Department of Internal Medicine, Faculty of Medical Sciences, Lebanese University, Hadath, Lebanon, ul.edu.lb; ^3^ Department of Internal Medicine, Faculty of Medicine, Beirut Arab University, Beirut, Lebanon, bau.edu.lb; ^4^ Department of Cardiology, Hammoud Hospital University Medical Center, Saida, Lebanon, hammoudhospital.com

**Keywords:** cardio-hepatorenal syndrome, challenge, echocardiogram, infective endocarditis, viral infection

## Abstract

Infective endocarditis (IE) imposes a challenge in clinical practice, often demanding rapid and precise identification of the causative agent to guide tailored therapeutic interventions. However, a subset of cases presents an elusive diagnostic dilemma with culture‐negative results, thereby complicating the management strategy. This case report sheds light on a particularly rare instance of culture‐negative IE in a 21‐year‐old previously healthy male who presented with dyspnea and jaundice, emphasizing the scarcity of such cases in the existing medical literature. Through a detailed examination of clinical manifestations, diagnostic modalities, and treatment outcomes, we aim to contribute valuable insights into the nuanced aspects of culture‐negative IE. Furthermore, this report underscores the imperative need for clinicians to maintain a high index of suspicion for IE, even in the absence of positive cultures, to ensure timely and appropriate management for improved patient prognosis.

## 1. Introduction

Infective endocarditis (IE) is an inflammation of the endocardium and heart valves due to bacterial, fungal, or, less commonly, viral infections [1]. Although relatively rare, IE remains a serious cardiac condition, particularly in patients with predisposing risk factors or comorbidities. Its clinical presentation—acute, subacute, or chronic—varies depending on the causative organism [1, 2]. Diagnosis is based on clinical presentation, laboratory results, and imaging findings, using the modified Duke criteria [1, 2].

From a microbiological perspective, most cases of IE are due to *Staphylococcus aureus*, viridans group streptococci, or enterococci. However, up to 20% of cases may be culture‐negative, often due to prior antibiotic therapy or infection with fastidious organisms such as *Coxiella burnetii*, *Bartonella* species, and fungi [3–5].

Delayed diagnosis of IE can lead to severe complications, particularly valvular damage that, if untreated, may progress to congestive heart failure (CHF)—the most critical complication, significantly affecting prognosis [6]. CHF can result in cardio‐hepatorenal syndrome (CHRS), a serious condition causing multi‐organ dysfunction involving the heart, liver, and kidneys [7, 8]. Valvular disease‐induced CHF can impair liver and kidney function due to reduced cardiac output, sometimes causing reversible liver and kidney ischemia [7, 8]. The complexity of CHRS, marked by symptoms of decompensation and altered liver function tests, can lead to misdiagnosis of hepatic viral infections, drug‐induced disorders, or prerenal causes [7, 8].

We report an uncommon presentation of culture‐negative IE in a previously healthy 21‐year‐old male who initially presented with dyspnea and jaundice.

## 2. Case Presentation

A previously healthy 21‐year‐old male presented with 1 week of dyspnea at rest and jaundice. His symptoms had begun 1 month earlier with exertional dyspnea that progressively worsened, accompanied by dark urine, intermittent chills, and one undocumented fever episode.

Initially, he was misdiagnosed at a peripheral hospital in Lebanon with viral Hepatitis A and pneumonia and treated with intravenous hydration and levofloxacin. No laboratory confirmation of Hepatitis A was available, and no causative organism for pneumonia was identified. His condition deteriorated, prompting transfer. Notably, he had a prior hospitalization 1 year earlier for pneumonia, managed with broad‐spectrum ertapenem, though no causative organism had been identified. He denied chest pain, palpitations, diaphoresis, intravenous drug use, sexual exposure, or recent COVID‐19 infection/exposure.

**Figure 1:**
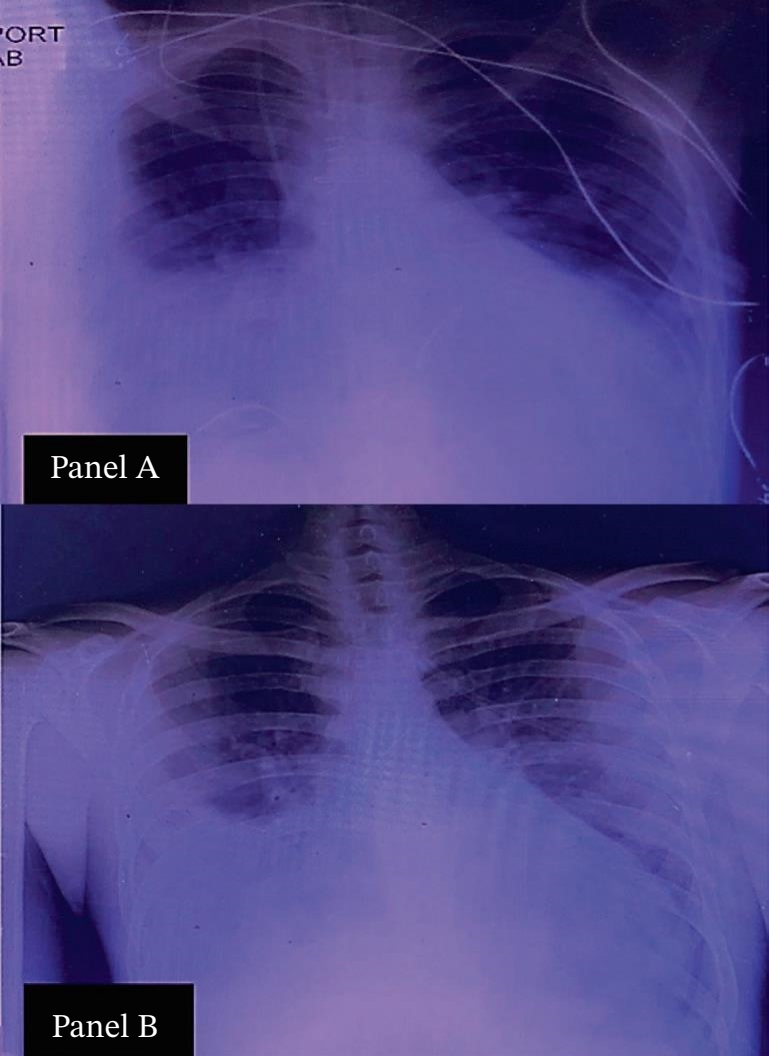
Chest radiographs demonstrating pulmonary congestion and its resolution, (a) portable chest radiograph obtained on admission showing diffuse pulmonary congestion and cardiomegaly and (b) follow‐up chest radiograph at discharge demonstrating resolution of pulmonary congestion and improved lung fields.

On admission, he was in moderate respiratory distress but hemodynamically stable with an SpO₂ of 98% on facemask. Physical examination revealed jaundice, icteric sclerae, distended jugular veins, and a pansystolic murmur. Lung auscultation demonstrated diffuse crepitations, and abdominal examination confirmed hepatomegaly. No ascites or peripheral edema was present. Chest X‐ray showed pulmonary congestion, which improved and subsequently resolved during his hospital stay (Figure 1).

Laboratory results indicated elevated BUN, creatinine, uric acid, and abnormal liver enzymes Table (1). Blood cultures were consistently negative. Full laboratory data are provided in Appendix A of the Supporting Information 1

**Table 1:** Summary of Laboratory Results.

Lab Test	Value
White blood cells (WBCs)	13,000 cells/*μ*L
Neutrophils	82%
Hemoglobin	12 g/dL
Platelets	269 × 10^9^/L
Blood urea nitrogen (BUN)	99 mg/dL
Creatinine	1.6 mg/dL
Uric acid	16 mg/dL
Serum glutamic oxaloacetic transaminase (SGOT)	163 U/L
Serum glutamic pyruvic transaminase (SGPT)	150 U/L
Gamma‐glutamyl transferase (GGT)	65 U/L
Creatine phosphokinase (CPK)	359 U/L
Total bilirubin	11.6 mg/dL
Direct (conjugated) bilirubin	6.5 mg/dL
Indirect (unconjugated) bilirubin	5.1 mg/dL
HBsAg	Negative
Anti‐HAV IgM antibodies	Nonreactive (0.86)
HAV Total IgG Antibodies	Immune (13.12)
ANCA‐P	Negative
ANCA‐C	Negative

A computed tomography (CT) scan performed on Day 3 revealed moderate hepatomegaly (17.5 cm midclavicular line) and severe cardiomegaly, supporting the suspicion of CHRS.

Echocardiography showed multiple valve issues, an enlarged left ventricle with an ejection fraction (EF) of 50%, and a para‐aortic cavity due to left cusp destruction, causing severe aortic regurgitation. A transesophageal echocardiogram (TEE) confirmed moderate left ventricular dilation (EF 45%–50%), Grade II mitral and tricuspid regurgitation, a bicuspid aortic valve with possible vegetations, and Grade IV aortic insufficiency with mild pericardial effusion.

**Figure 2:**
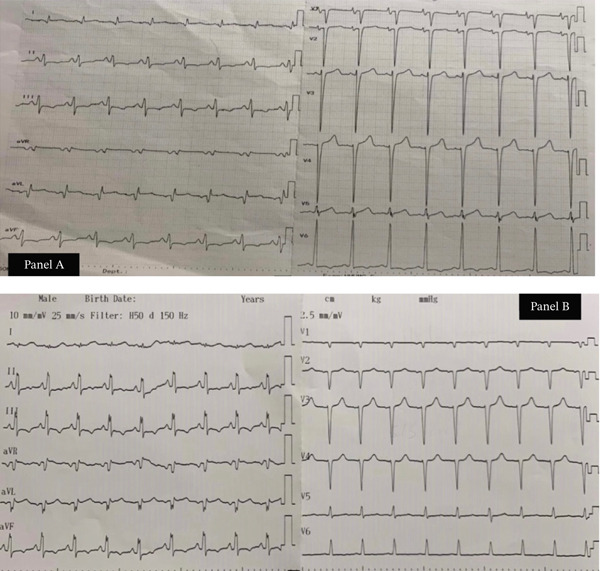
Serial electrocardiograms demonstrating preoperative and postoperative findings, (a) 12‐lead ECG on admission (Day 1) showing sinus rhythm, voltage criteria for left ventricular hypertrophy (LVH), and lateral T‐wave inversions consistent with left ventricular strain pattern and (b) follow‐up ECG on Day 10 demonstrating sinus tachycardia with partial resolution of lateral T‐wave inversions, consistent with improved left ventricular repolarization after surgical correction of aortic regurgitation.

Representative electrocardiograms (ECGs) obtained on admission (Day 1) and after surgery (Day 10) are shown in Figure 2.

Initial management included oxygen therapy, intravenous diuretics, and empiric broad‐spectrum antibiotics. Cardiology and cardiac surgery teams recommended urgent surgery due to severe aortic regurgitation with heart failure unresponsive to medical therapy.

The patient underwent complex multivalve and aortic surgery. The ascending aorta and aortic valve were replaced with reimplantation of the coronary ostia using a Freestyle No. 25 porcine valve‐conduit. The mitral valve was repaired with annuloplasty using a Physio Edwards No. 32 ring, and the tricuspid valve was repaired with annuloplasty using a Medtronic No. 32 ring. Note that TEE annular measurements used to select the No. 32 mitral and tricuspid annuloplasty rings were not available, as the corresponding digital imaging records were irretrievably lost due to system failures associated with the recent conflict in Lebanon. This limitation is acknowledged. Cardiopulmonary bypass (CPB) time was 110 min, and aortic cross‐clamp (ACC) time was 80 minutes. Intraoperative biopsy showed fibrotic and myxoid changes without microorganisms identified on standard stains.

Postoperatively, the patient′s symptoms improved significantly. At 1‐month follow‐up, echocardiography demonstrated an EF of 55%, with normalization of bilirubin and creatinine. He was discharged on an ACE inhibitor, diuretic, and antiplatelet therapy, with instructions for close follow‐up.

## 3. Discussion

This case illustrates the diagnostic and therapeutic challenges of culture‐negative IE in a young, previously healthy male who developed severe valvular dysfunction and CHRS.

Culture‐negative IE occurs in up to 20% of cases and is often attributed to prior antibiotic exposure or infection with fastidious organisms such as *Coxiella burnetii*, *Bartonella* species, *Tropheryma whipplei*, or fungi [5, 9]. In addition to careful clinical evaluation and echocardiography, it can be investigated using a range of advanced methods, including serology for atypical organisms, broad‐range 16S rRNA polymerase chain reaction (PCR), and targeted molecular assays on excised valve tissue. Incorporating these tests into the diagnostic strategy increases sensitivity and helps identify pathogens that escape standard blood cultures [5]. Our patient received levofloxacin before blood cultures were obtained, which may have contributed to negative results. Advanced diagnostic methods such as PCR and serology were not available at our institution, a limitation we now acknowledge. Their absence in our case underscores the diagnostic challenges faced in resource‐limited settings.

Noninfectious causes of valvular damage were also considered. Autoimmune panels, including ANCA‐C and ANCA‐P, were negative, and histopathology did not demonstrate features consistent with rheumatic or Libman–Sacks endocarditis.

The presence of a bicuspid aortic valve is noteworthy. This congenital anomaly predisposes patients to IE due to abnormal hemodynamics and structural vulnerability [10]. In this case, the rapid progression to severe regurgitation and left ventricular dilation reflects the aggressive course of left‐sided IE.

We diagnosed CHRS based on the triad of cardiac dysfunction (severe AR, LV dilation, and CHF), hepatic dysfunction (jaundice, hepatomegaly, and elevated bilirubin), and renal dysfunction (elevated creatinine and BUN). Viral hepatitis was ruled out by negative serologies, and drug‐induced injury was excluded by history [11].

Complications of IE are varied and include heart failure, systemic embolization, intracardiac abscess, conduction abnormalities, and multi‐organ dysfunction [3]. In our patient, severe aortic and mitral regurgitation progressed to CHF, which in turn precipitated CHRS, demonstrating the systemic impact of delayed recognition. The need for urgent surgical intervention reflects how rapidly IE can evolve to life‐threatening complications in young patients, even in the absence of preexisting comorbidities.

Timely surgical intervention was essential. Surgery was indicated due to severe AR with CHF unresponsive to medical therapy. Repairs included mitral valvuloplasty, tricuspid annuloplasty, and the replacement of the ascending aorta and aortic valve with a Freestyle No. 25 procrine valve‐conduit. The patient′s rapid postoperative improvement highlights the role of early surgery in IE complicated by CHF [12].

Comparison with the literature shows that reports of CNIE in young adults without comorbidities are rare, and outcomes are highly variable depending on surgical timing. Our case adds to this limited body of evidence by illustrating recovery following urgent multivalve surgery [9].

## 4. Conclusion

This case highlights the importance of maintaining a high index of suspicion for culture‐negative IE, particularly in young patients presenting with multi‐organ dysfunction. Early recognition of complications such as CHF and CHRS, combined with timely surgical intervention, is crucial for improving outcomes.

### 4.1. Limitations

Our study faced several limitations, including the lack of complete medical records from the patient′s previous admission, which limited our ability to confirm the causative organism of the earlier pneumonia. Additionally, advanced microbiological methods (PCR, serology, and 16S rRNA sequencing) were not available at our institution, preventing further pathogen identification [5]. A portion of the patient′s imaging data—including the original high‐resolution chest radiographs, detailed TEE annular measurements, and other archived diagnostic files—could not be retrieved because the hospital′s electronic system experienced irreversible data loss during the recent conflict in Lebanon. Despite these limitations, this case underscores the value of thorough clinical evaluation and highlights gaps in diagnostic resources.

## Funding

No funding was received for this manuscript.

## Ethics Statement

Ethical approval was not required for this single‐patient case report, in accordance with institutional and national regulations.

## Consent

Written informed consent for publication, including clinical details and imaging, was obtained from the patient.

## Conflicts of Interest

The authors declare no conflicts of interest.

## Supporting information


**Supporting Information** Additional supporting information can be found online in the Supporting Information section. Appendix A: Summary of Laboratory Results of the patient over 10 days from first admission.

## Data Availability

The original contributions presented in the study are available upon request.
