# Supplementary material for: Culture‐Negative Infective Endocarditis Presenting With Cardio‐Hepatorenal Syndrome in a 21‐Year‐Old Patient: A Case Report of Diagnostic Workup and Management
Source: Case Rep Cardiol. 2026 Mar 9;2026:7676322. doi: 10.1155/cric/7676322 (PMC12968723; doi:10.1155/cric/7676322)
Supplement: Supplementary file 1 — Supporting Information Additional supporting information can be found online in the Supporting Information section. Appendix A: Summary of Laboratory Results of the patient over 10 days from first admission. [file CRIC-2026-7676322-s001.docx]

| **Lab Tests** | **Day 1** | **Day 2** | **Day 3** | **Day 4** | **Day 5** | **Day 6** | **Day 7** | **Day 8** | **Day 9** | **Day 10** |
| --- | --- | --- | --- | --- | --- | --- | --- | --- | --- | --- |
| White Blood Cells (x10^3^/μL) | 13 | 12.4 | 9.7 | 10 | 10 | 12 | 13 | 14 | 39 | 20 |
| Neutrophils (%) | 82 | 83 | 83 | 84 | 85 | 85 | 85 | 86 | 90 | 90 |
| Hemoglobin (g/dl) | 12 | 10.1 | 10.5 | 10.2 | 10.3 | 9.9 | 9.5 | 9.4 | 9.5 | 7.8 |
| Mean Corpuscular Volume (MCV) (fL) | 89 | 88 | 83 | 83 | 85 | 87 | 88 | 87 | 88 | 88 |
| Platelets (x10^3^/μL) | 269 | 246 | 229 | 217 | 249 | 302 | - | 292 | 319 | 277 |
| Blood Urea Nitrogen (mg/dl) | 99 | 99 | 74 | 34 | 18 | 21 | 20 | 20 | 20 | 25 |
| Creatinine (mg/dl) | 1.6 | 1.5 | 1.4 | 0.9 | 0.7 | 0.7 | 0.6 | 0.6 | 0.6 | 0.6 |
| Uric Acid (mg/dl) | 16 | - | - | 4.8 | 3.3 | - | - | - | - | - |
| Sodium (Na) (mEq/L) | 127 | 129 | 130 | 133 | 134 | 133 | 135 | 133 | 136 | 141 |
| Potassium (K) (mEq/L) | 5.1 | 5.1 | 5.2 | 5.2 | 5.3 | 5.2 | 5.2 | 4.8 | 4.5 | 4.4 |
| Chloride (Cl) (mEq/L) | 92 | 95 | 96 | 100 | 102 | 102 | 101 | 103 | 100 | 104 |
| International Normalized Ratio (INR) | 2.12 | - | - | - | 1.59 | 1.75 | - | 1.86 | 1.57 | 1.27 |
| Partial Thromboplastin Time (PTT) (seconds) | 28 | - | - | - | 29 | 29 | - | 27 | 28 | 26 |
| Total bilirubin (mg/dl) | 11.6 | - | 9 | 7.4 | - | 5.5 | 5.6 | 5.6 | 6.7 | 5.8/3.7 |
| Direct (conjugated) bilirubin (mg/dl) | 6.5 |  | 6 | 4.8 |  | 3.7 | 3.6 | 3.8 | 4.4 | 3.7 |
| Indirect (unconjugated) bilirubin (mg/dl) | 5.1 |  | 3 | 2.6 |  | 1.8 | 2 | 1.8 | 2.3 | 2.1 |
| Alkaline Phosphatase (U/L) | 78 | - | 76 | - | - | 143 | 95 | 123 | - | - |
| Serum Glutamic Oxaloacetic Transaminase (SGOT) (U/L) | 163 | - | 170 | - | - | 95 | 173 | 395 | 567 | 243 |
| Serum Glutamic Pyruvic Transaminase (SGPT) (U/L) | 150 | - | 111 | 93 | 83 | 97 | 115 | 233 | 402 | 213 |
| Gamma-Glutamyl Transferase (GGT) (U/L) | 65 | - | 59 | - | - | 449 | 455 | 199 | 316 | - |
| Creatine Phosphokinase (CPK) (mcg/L) | 359 | - |  | - | 568 | - | - | 103 | - | - |
| C-Reactive Protein (CRP) (mg/L) | 351 | - | 269 | 225 | 198 | - | 155 | 139 | 109 | - |
| -**Day 1:** smear normocytic normochromic RBCs slight polychromasia target and cigar-shaped cells are seen.  -**Day2:** Urine Analysis: 1-2 WBCS; 12-14 RBCs; Specific gravity: 1.01; Monomorphic fresh RBCs; No RBCs casts.  -**Day3:** Coombs Direct: positive indirect negative; HIV: 0.19; CMV IgG: reactive> 250; CMV IgM: 0.7; EBV IgG: 2.6; EBV IgM: 1.5; Procalcitonin: 7.35; IgM Abs for SARS-CoV2: 0.28(A); IgG Abs for SARS-CoV2: 0.29(A)  **- Day4:** ANCA-P: negative; ANCA-C: negative  -**Day6:** HBsAg: negative; Anti-HAV IgM antibodies: 0.86 (Non-reactive); HAV Total IgG antibodies: 13.12 (immune to hepatitis A from wither past infection or vaccination)  ***Note:***   - Total bilirubin: this is the combined measure of both direct and indirect bilirubin. - Direct (conjugated) bilirubin: this is the form of bilirubin that has been processed by the liver and is ready to be excreted in the bile.   Indirect (unconjugated) bilirubin: this is the form of bilirubin that has not yet been processed by the liver. | | | | | | | | | | |

**Appendix A:** Summary of Laboratory Results of the patient over 10 days from first admission
